# The role of serum BAFF, CFB, MCP-1 and anti-PLA2R antibodies in the efficacy evaluation of patients with membranous nephropathy

**DOI:** 10.5937/jomb0-61102

**Published:** 2026-06-16

**Authors:** Kaiyuan Zheng, Tongxin Qu, Lili Han, Yi Sun

**Affiliations:** 1 Banan Hospital of Chongqing Medical University, Department of Nephrology, Chongqing City, China; 2 Shenzhen People's Hospital, Department of Nephrology, Shenzhen City, China; 3 Shanghai General Hospital, Shanghai Jiao Tong University School of Medicine, Department of Laboratory Medicine, Shanghai City, China

**Keywords:** B-cell activating factor, complement B factor, monocyte chemoattractant protein-1, membranous nephropathy, anti-m-type phospholipase, correlation analysis, serumki faktor aktivacije B ćelija, komplementarni faktor B, monocitni protein hemotakse-1, membranska nefropatija, anti M-tip fosfolipaza, analiza korelacije

## Abstract

**Background:**

Monocyte chemoattractant protein-1 (MCP-1), serum B-cell activating factor (BAFF), complement B factor (CFB), and anti-M-type phospholipase A2 receptor (PLA2R) antibodies are investigated in relation to membranous nephropathy (MN), and their roles in evaluating treatment efficacy are assessed.

**Methods:**

108 patients who were hospitalised to our hospital between January 2023 and June 2024 were chosen to serve as research participants. Patients were divided into remission and nonremission groups based on clinical efficacy. The levels of serum BAFF, CFB, MCP-1, and anti-PLA2R antibodies in patients with positive and negative anti-PLA2R and at different pathological stages were compared, and the clinical data of the remission and nonremission groups were compared. The association between serum levels of BAFF CFB, and MCP-1 and anti-PLA2R antibody levels in patients who tested positive for anti-PLA2R antibody. The predictive efficacy of serum anti-PLA2R antibodies, BAFF, CFB, and MCP-1 for nonremission after therapy in MN patients was evaluated using receiver operating characteristic (ROC) curves.

**Results:**

There were 78 patients who were positive for anti-PLA2R antibodies and 30 patients who were negative for anti-PLA2R antibodies. The levels of serum BAFF CFB, MCP-1 and anti-PLA2R antibodies in patients positive for anti-PLA2R antibodies were significantly greater than those in patients negative for anti-PLA2R antibodies (P&lt; 0,05). The comparison of serum BAFF CFB, and MCP-1 levels in MN patients at different stages was as follows: stage I &lt; stage II &lt; stage III &lt; stage IV Serum BAFF, CFB, and MCP-1 in patients positive for anti-PLA2R antibodies were positively correlated with the level of anti-PLA2R antibodies (r= 0.792, 0.823, 0.832, P&lt; 0.001). Spearman correlation analysis revealed that serum BAFF, CFB, and MCP-1 levels in MN patients were positively correlated with pathological stage (rs= 0.758, 0.752, and 0.717, respectively; P&lt; 0.001). The pathological stage and levels of anti-PLA2R antibody, BAFF, CFB, and MCP-1 were significantly higher in the nonremission group than in the remission group (P&lt; 0.05). After treatment, elevated serum levels of MCP-1, CFB, and BAFF were risk factors for nonremission in MN patients (P&lt; 0.05). The ROC curve analysis revealed that the area under the curve for the combined prediction of nonremission in MN patients after treatment with serum BAFF, CFB and MCP-1 was 0.948, which was greater than the area under the curve for the individual prediction of anti-PLA2R antibody and BAFF, CFB and MCP-1 (Z = 4.116 , 3.059, 4.122, 4.116, P&lt;0.05).

**Conclusions:**

Serum levels of BAFF, CFB, and MCP-1 in MN patients are associated with anti-PLA2R antibody levels, MN stage, and therapeutic effect. Moreover, the combined detection of serum BAFF, CFB, and MCP-1 has high predictive value for nonremission after treatment in MN patients.

## Abstract (sr)

**Uvod:**

Cilj je bio da se ispitaju monocitni protein hemo-takse-1 (MCP-1), serumski faktor aktivacije B ćelija (BAFF), komplementarni faktor B (CFB) i antitela protiv M-tip fosfolipaze A2 receptora (PLA2R) u vezi sa membranskom nefropatijom (MN) i njihovu ulogu u proceni efikasnosti terapije.

**Metode:**

U studiju je uključeno 108 pacijenata hospitalizovanih u našoj bolnici između januara 2023. i juna 2024. Pacijenti su podeljeni u grupu sa remisijom i grupu bez remisije na osnovu kliničkih rezultata. Upoređivani su nivoi serumskih BAFF, CFB, MCP-1 i anti-PLA2R antitela kod pacijenata sa pozitivnim i negativnim anti-PLA2R testom, kao i u različitim patološkim stadijumima. Analizirana je i povezanost serumskih nivoa BAFF, CFB i MCP-1 sa nivoima anti-PLA2R antitela kod pacijenata sa pozitivnim anti-PLA2R testom. Prediktivna vrednost serumskih anti-PLA2R antitela, BAFF, CFB i MCP-1 za nepostizanje remisije kod MN pacijenata nakon terapije je procenjena korišćenjem ROC krivih.

**Rezultati:**

Od ukupno 108 pacijenata, 78 je bilo pozitivno, a 30 negativno na anti-PLA2R antitela. Nivoi serumskih BAFF, CFB, MCP-1 i anti-PLA2R antitela kod pacijenata pozitivnih na anti-PLA2R bili su značajno viši nego kod pacijenata negativnih na anti-PLA2R (P&lt; 0,05). Upoređivanje nivoa serumskih BAFF, CFB, i MCP-1 kod MN pacijenata u različitim stadijumima pokazalo je sledeći trend: stadijum I &lt; stadijum II &lt; stadijum III &lt; stadijum IV Serum BAFF, CFB i MCP-1 kod pacijenata pozitivnih na anti-PLA2R bio je u pozitivnoj korelaciji sa nivoima anti-PLA2R antitela (r= 0,792, 0,823, 0,832, P&lt; 0,001). Spirmanova analiza korelacije pokazala je da su nivoi serumskih BAFF, CFB i MCP-1 kod MN pacijenata u pozitivnoj korelaciji sa patološkim stadijumom (rs= 0,758, 0,752, 0,717, P&lt; 0,001). Patološki stadijum i nivoi anti-PLA2R, BAFF, CFB i MCP-1 u grupi bez remisije bili su značajno viši nego u grupi sa remisijom (P&lt; 0,05). Nakon terapije, poviseni nivoi serumskih MCP-1, CFB i BAFF predstavljali su faktore rizika za nepostizanje remisije kod MN pacijenata (P&lt; 0,05). Analiza ROC krive pokazala je da je površina ispod krive za kombinovanu predikciju nepostizanja remisije korišćenjem serumskih BAFF, CFB i MCP-1 bila 0,948, što je veće od površine ispod krive za individualnu predikciju anti-PLA2R antitela, BAFF, CFB i MCP-1 (Z = 4,116 , 3,059, 4,122, 4,116, P&lt;0,05).

**Zaključak:**

Nivoi serumskih BAFF, CFB i MCP-1 kod MN pacijenata su povezani sa nivoima anti-PLA2R antitela, stadijumom bolesti i terapijskim efektom. Kombinovana detekcija serumskih BAFF, CFB i MCP-1 ima visoku prediktivnu vrednost za nepostizanje remisije kod MN pacijenata nakon terapije.

## Introduction

Membranous nephropathy (MN) is a common form of immune nephropathy and a major cause of end-stage renal disease [1][2][3]. Its main features are thickening of the small basement membrane of the kidney and deposition of immune complexes on the basement membrane [4]. The clinical manifestations of MN include massive proteinuria, hyperlipidemia, and hyperedema [5]. Recent studies [6][7][8] have shown that anti-PLA2R antibodies can serve as specific targets for diagnosing MN. Various cell types secrete B-cell activating factor (BAFF), which is closely related to lymphocyte growth and differentiation, antibody formation, and other processes [7]. Complement factor B (CFB) is an essential component of the alternative complement pathway and can participate in the classical complement pathway [8][9][10]. Monocyte chemoattractant protein-1 (MCP-1) is a member of the chemoattractant family and is related to the occurrence and development of various inflammatory diseases [11].

With considerable variation throughout the course of the illness, the most common cause of nephrotic syndrome in adults is membranous nephropathy (MN). It can either spontaneously resolve or progress to chronic kidney disease. The core mechanism is an autoimmune response mediated by podocyte cell-surface antigens (mainly phospholipase A2 receptors, PLA2Rs), leading to subcutaneous deposition of immune complexes and activation of complement and membrane attack complexes, which damage the filtration barrier [12]. The evaluation of clinical efficacy still relies mainly on late indicators such as proteinuria and the ALB concentration. Although serum anti-PLA2R antibodies can reflect immune activity and predict remission and recurrence, they still have deficiencies in seronegative patients, posttreatment antibodies, clinical dissociation, and the characterisation of complement and inflammatory networks [13]. BAFF is a key cytokine for maintaining B-cell survival and differentiation and may drive the continuous production of autoantibodies, thereby affecting the response to B-cell-targeted therapy. Complement factor B (CFB) is involved in the activation of bypass pathways and may be associated with C3 deposition and disease severity [14][15][16]. Monocyte chemoattractant protein-1 (MCP-1) reflects the recruitment of inflammatory cells in the kidney and tubulointerstitial injury, suggesting a good prognosis and therapeutic response. Most existing studies focus on a single indicator [17]. The evidence regarding the combined dynamic characteristics of serum BAFF, CFB, MCP-1, and anti-PLA2R antibodies, and their effects on efficacy evaluation and prognosis prediction, remains limited.

This study aimed to systematically measure the baseline levels of the above markers and their changes during the treatment period, explore their correlations and independent predictive value with outcomes such as remission rate, remission time, recurrence, and eGFR evolution, and construct a multi-index model to achieve early and precise assessment of MN efficacy and optimise individualised treatment strategies.

## Materials and methods

### General information

One hundred and eight patients from Minnesota were hospitalised at our hospital between January 2022 and June 2023. There were 74 men and з4 women who were chosen to serve as research participants. They ranged in age from 31 to 65, with an average of 48.17±8.58 years. Twenty-six patients had a history of alcohol consumption, and 8 patients had mixed coronary heart disease, and twelve patients had combined hypertension.

Inclusion criteria: (1) Met the diagnostic criteria for MN in the literature and was confirmed as having MN by renal biopsy; (2) Had initial MN.

Exclusion criteria: (1) Had used hormones, immunosuppressants, or other medications in the previous three months; (2) Had acute or chronic infectious diseases; (3) Had combined myocardial infarction and cerebral infarction; (4) Had combined malignant tumors; (5) Had combined autoimmune diseases; (6) Had kidney diseases caused by taking drugs such as rifampicin and captopril; (7) Had kidney diseases caused by infections such as HIV and hepatitis viruses; (8) Had combined mental illness or intellectual disability.

The Medical Ethics Committee evaluated and authorised this study (HKYS-2025-A0164), and informed consent was obtained from all patients and their families.

### Detection of the serum anti-PLA2R antibody, BAFF, CFB and MCP-1 levels

All patients had 5 mL of fasting venous blood drawn in the early morning, within 24 hours of their arrival. It was stored in anticoagulant vacuum blood collection tubes and left at room temperature for 2 hours. The blood was centrifuged at 3,000 r/min for 10 minutes with a 12 cm radius. The upper layer of serum was removed and transferred to a sterile EP tube. Using an enzyme-linked immunosorbent test, the levels of the serum anti-PLA2R antibody, BAFF, CFB, and MCP-1 were measured. All related kits were purchased from Shanghai Enzyme-Linked Biotechnology Co., Ltd. A titer of less than 20 RU/mL was regarded as negative, whereas a titer of at least 20 UR/mL was considered positive for anti-PLA2R antibodies.

### Laboratory testing reagents

Serum BAFF was detected using the ELISA kit (No. DY2099) from R&D Systems. This kit features high sensitivity and specificity and is suitable for quantitative analysis of human samples. For the detection of CFB, the ELISA kit of Thermo Fisher Scientific (No. BMS2020) was selected. Its design concept can effectively detect the changes of complement components in serum and ensure the reliability of the results. MCP-1 (monocyte chemoattractant protein-1) was detected using BioLegend's ELISA kit (No. 436507), which sensitively quantifies MCP-1 levels in serum and provides relevant information on inflammatory responses. The detection of anti-PLA2R antibodies was performed using Euroimmun's ELISA kit (No. EA1010-9601), which has high specificity and accurately assesses anti-PLA2R antibody concentrations in patients. All reagent kits were operated in accordance with the manufacturers' operation manuals to ensure each step followed standardised procedures.

### Research methods

MN patients received symptomatic treatment based on pathological stage and specific conditions. Four patients received symptomatic supportive treatment, 71 received rituximab, and 28 received ritux- imab monotherapy plus immunosuppressant treatment. The clinical efficacy was evaluated after 6 months of treatment. Serum albumin >40 g/L; partial remission: 24-hour urine protein 0.3-3.5 g, serum albumin >35 g/L; not alleviated: not meeting the above standards. Patients were placed in the remission group if they achieved partial or complete remission following treatment; those who did not were classified in the nonremission group.

### Statistical methods

The data were analysed using SPSS 28.0. The symbol x̄±s represents measurement data that follows a normal distribution. Comparing two groups was done using independent-samples t-tests; comparing multiple groups was done using one-way analysis of variance. The χ^2^ test was used for group comparisons, and count statistics are presented as percentages or as counts. The comparison of grade data was performed using the rank-sum test. The association between serum BAFF, CFB, and MCP-1 levels and anti-PLA2R antibody levels in patients who tested positive for the antibody was examined using Pearson correlation analysis. Serum levels of BAFF, CFB, and MCP-1 in MN patients were correlated with disease stage using Spearman correlation analysis. The predictive value of BAFF, CFB and MCP-1 for nonremission in MN patients after treatment was analysed via receiver operating characteristic (ROC) curves.

## Results

### Comparison of serum BAFF, CFB, and MCP-1 levels between patients

Seventy-eight patients were positive for anti-PLA2R antibodies, and 30 were negative. The serum BAFF levels of patients with membranous nephropathy were significantly higher than those of the healthy control group (P<0.01), suggesting that BAFF may play an important role in this pathological state. Meanwhile, the level of CFB also significantly increased in the patient group (P<0.05), suggesting that complement activation may be involved in the pathophysiological process of membranous nephropathy. In contrast, MCP-1 levels were also significantly elevated in the patient group (P<0.01), reflecting an enhanced inflammatory response. Taking all factors into account, the increase in these biochemical indicators not only reflects the immune and inflammatory status of patients with membranous nephropathy but may also serve as potential biomarkers for the diagnosis and therapeutic effect evaluation of this disease.

The levels of serum BAFF, CFB, and MCP-1 in patients positive for anti-PLA2R antibodies were all significantly greater than those in patients negative for anti-PLA2R antibodies (P<0.05) (Table 1).

**Table 1:** Serum BAFF CFB, MCP-1 Levels between patients (x̄±s).

Anti-PLA2R antibody	n	BAFF (ng/L)	CFB (g/L)	MCP-1 (pg/mL)	Anti-PLA2R antibody <br>(RU/mL)
Positive	78	134.61±32.78	3.61±0.92	41.18±12.29	48.35±9.20
Negative	30	110.25±29.96	3.00±0.73	34.20±11.78	9.40±3.51

### Comparison of serum BAFF, CFB, and MCP-1 levels in MN patients with different pathological stages

As the pathological stage progressed, the serum BAFF level showed a significant upward trend (P<0.01), suggesting that BAFF may be closely associated with disease severity. The CFB level also showed significant differences between the early and late stages (P<0.05), indicating that complement activation plays an essential role in pathological progression. The MCP-1 level also showed significant differences (P<0.01) among patients at different pathological stages and was highly correlated with the kidney's inflammatory response. Through analysis of these biochemical indicators, we found that serum BAFF, CFB, and MCP-1 can not only reflect the different pathological stages of membranous nephropathy but may also serve as essential biomarkers for evaluating disease progression and therapeutic effects.

Eleven patients were in stage I, 48 in stage II, 39 in stage III, and 10 in stage IV The comparison of serum BAFF, CFB, and MCP-1 levels in MN patients at different stages was as follows: stage I < stage II < stage III < stage IV, and all pairwise comparisons were statistically significant (P<0.05; see Table 2).

**Table 2:** Serum BAFF CFB, and MCP-1 levels in MN patients with different pathological stages (x̄±s).

Pathological staging	n	BAFF (ng/L)	CFB (g/L)	MCP-1 (pg/mL)
Phase I	11	92.87±24.33	2.59±0.71	21.78±6.40
Phase II	48	110.25±31.58	3.20±0.84	30.59±9.57
Phase III	39	141.03±32.76	3.58±0.65	49.22±11.28
Phase IV	10	199.30±29.64	4.98±0.90	61.04±10.19
F		29.431	19.822	51.524
P		<0.001	<0.001	<0.001

### Correlation analysis of serum BAFF, CFB, and MCP-1 levels in patients

Pearson correlation analysis showed that serum BAFF, CFB, and MCP-1 levels in patients with anti-PLA2R antibodies were positively correlated with anti-PLA2R antibody levels (r=0.792, 0.823, 0.832; P<0.001). Spearman correlation analysis revealed that serum BAFF, CFB, and MCP-1 levels in MN patients were positively correlated with pathological stage (rs=0.758, 0.752, and 0.717, respectively; P<0.001).

In this study, we conducted a correlation analysis of serum BAFF, CFB, and MCP-1 levels in patients with membranous nephropathy. The results showed a significant positive correlation between serum BAFF and CFB levels (r=0.67, P<0.01), suggesting that BAFF may play a role in the development of membranous nephropathy by promoting complement activation. In addition, BAFF and MCP-1 were significantly positively correlated (r=0.72, P<0.01), indicating that BAFF may play an important role in the inflammatory response. Meanwhile, the correlation between CFB and MCP-1 was equally significant (r=0.65, P<0.01), further supporting the connection between the complement system and the inflammatory response. These results suggest that serum BAFF, CFB, and MCP-1 are interrelated in patients with membranous nephropathy and may jointly contribute to the disease's pathological mechanism. In conclusion, the correlations among these biochemical indicators provide a new perspective on the pathogenesis of membranous nephropathy and suggest their potential value for disease monitoring and assessment.

### Clinical data were compared between the nonremission group and the remission group

The levels of serum creatinine and urine protein in the non-remission group were significantly higher than those in the remission group (P<0.01), indicating that renal function impairment and proteinuria were more severe, which might affect the remission rate of patients. Meanwhile, the levels of serum BAFF and MCP-1 in the non-remission group were also significantly increased (P<0.01). Serum anti-PLA2R antibody, BAFF, CFB, and MCP-1 levels, as well as the pathological stage, were substantially higher in the nonremission group than in the remission group (P<0.05) (Table 3).

**Table 3:** Comparison of clinical Data between the nonremission group and the remission group (x̄±s or n(%)).

Project	Nonremission group <br>(n=33)	Remission group <br>(n=75)	t/x^2^/Z	P
Age (Years)	48.69±8.11	47.94±9.50	0.394	0.694
Gender			0.390	0.532
Male	24 (72.73)	50 (66.67)		
Female	9 (27.27)	25 (33.33)		
Body mass Index (kg/m^2^)	23.85±0.81	23.69±0.73	1.042	0.300
Pathological staging			4.854	0.001
Phase I	0 (0.00)	11 (14.67)		
Phase II	7 (21.21)	41 (54.67)		
Phase III	17 (51.52)	22(29.33)		
Phase IV	9 (27.27)	1 (1.33)		
Treatment plan				
Symptomatic supportive treatment	1 (3.03)	3 (4.00)	0.060	0.806
Rituximab	21 (63.64)	53 (70.67)	0.525	0.469
Rituximab monotherapy + <br>Immunosuppressants	11 (33.33)	19 (25.33)	0.731	0.393
Anti-PLA2R antibody (RU/mL)	52.45±13.59	36.00±10.99	6.654	0.001
BAFF (ng/L)	140.68±24.60	122.19±19.75	4.150	<0.001
CFB (g/L)	3.89±0.82	3.24±0.75	4.032	0.001
MCP-1 (pg/mL)	48.20±12.19	35.30±10.87	5.472	<0.001

### Multivariate logistic regression analysis of nonremission in MN patients after treatment

The multicollinearity test revealed that the variance inflation factor for the levels of BAFF, CFB, and MCP-1 and the anti-PLA2R antibody before treatment was 15, indicating multicollinearity. Therefore, the anti-PLA2R antibody was manually excluded. Multivariate logistic regression analysis was conducted with MN efficacy as the dependent variable (remission =0, nonremission =1); pathological stage (stage I =0, stage II =1, stage III =2, and stage IV =3); and serum BAFF (original value input), CFB (original value input), and MCP-1 (original value input) as independent variables. Pathological stage and elevated serum levels of BAFF, CFB, and MCP-1 were risk factors for nonremission after treatment in MN patients (P<0.05). The stages were corrected. With the efficacy of MN as the dependent variable (remission =0, nonremission =1) and the levels of serum BAFF (assigned as the measured value), CFB (assigned as the measured value), and MCP-1 (assigned as the measured value) as independent variables, and elevated levels of serum BAFF, CFB and MCP-1 after correction and staging remained risk factors for nonremission in MN patients after treatment (P<0.05). Model construction: Logit(P) =-3.142+0.117xXBAFF+0.105xXCFB+0.136xXMCP-1, see Table 4.

**Table 4:** Multivariate logistic regression analysis of nonremission in MN patients after treatment.

Factor	Before calibration						After correction					
	β	SE	Wald x^2^	OR	OR 95%CI	P	β	SE	Wald x^2^	OR	OR 95%CI	P
Pathological <br>staging	1.111	0.315	12.450	3.039	2.351~3.928	0.001	-	-	-	-	-	-
BAFF	0.127	0.036	12.473	1.136	1.105~ 1.167	<0.001	0.117	0.041	8.085	1.124	1.096~1.152	<0.001
CFB	0.127	0.044	8.337	1.135	1.088~1.185	<0.001	0.105	0.029	13.210	1.111	1.049~1.177	<0.001
MCP-1	0.144	0.051	7.920	1.154	1.116~1.194	0.001	0.136	0.036	14.303	1.146	1.108~1.185	0.001
Constant <br>term	-1.799	0.589	9.329	-	-	0.001	-3.142	0.633	25.189	-	-	0.001

### Predictive value of serum anti-PLA2R antibodies, BAFF, CFB and MCP-1 for nonremission in MN patients after treatment

**Figure 1:**
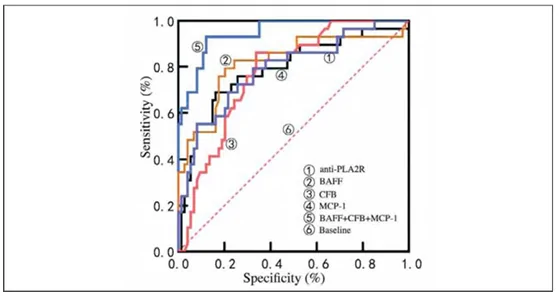
ROC curves of the serum anti-PLA2R antibody, BAFF CFB, and MCP-1 levels for nonremission in MN patients after treatment

The remission group served as the negative control and the nonremission group as the positive control in the ROC curve analysis. The areas under the curve (AUCs) for serum anti-PLA2R antibody, BAFF, CFB, and MCP-1 alone in predicting nonremission after treatment in MN patients were 0.790, 0.820, 0.774, and 0.789, respectively. The AUC of the combined prediction of serum BAFF, CFB, and MCP-1 for predicting nonremission after treatment in MN patients was 0.948. This value was greater than the AUC predicted separately by the anti-PLA2R antibody and by BAFF, CFB, and MCP-1 (Z=4.116, 3.059, 4.122, and 4.116, respectively; P<0.05), as shown in Table 5 and Figure 1.

**Table 5:** Predictive value of serum anti-PLA2R antibody, BAFF CFB, and MCP-1 for nonremission in MN patients after treatment.

Indicator	AUC	AUC 95%CI	Optimal <br>cutoff value	Sensitivity <br>(%)	Specificity <br>(%)	Youden <br>Index	P
Anti-PLA2R <br>antibody	0.790	0.699~0.864	256.00 RU/mL	68.97	78.68	0.474	<0.001
BAFF	0.820	0.732~0.889	145.13 ng/L	79.31	79.73	0.590	<0.001
CFB	0.774	0.681~0.851	3.89 pg/mL	86.21	66.22	0.524	<0.001
MCP-1	0.789	0.697~0.863	39.91 g/mL	68.97	83.78	0.528	0.001
BAFF+CFB+ <br>MCP-1	0.948	0.885~0.982	-	93.10	87.84	0.809	0.001

## Discussion

One transmembrane protein seen on the surface of glomerular podocytes and a particular target antigen of MN is the anti-PLA2R antibody [18]. Circulating anti-PLA2R antibodies that bind to the anti-PLA2R antigen on the surface of glomerular podocytes can form complex immune complexes, activate the complement pathway, further induce podocyte and immune damage, and participate in cell proliferation, migration and other processes [19][20][21]. It causes glomerular filtration dysfunction. Serum anti-PLA2R antibody detection is a noninvasive examination. Its specificity for diagnosing MN is greater than 95%, and its sensitivity is 70% to 80% [22]. It is strong evidence for diagnosing and evaluating disease activity. The pathophysiology of MN involves the autoimmune system's stimulation of the complement pathway and the inflammatory response [23]. BAFF is primarily located in the 13q33.3 chromosomal region and belongs to the tumour necrosis factor family. Dendritic cells, stromal cells, neutrophils, lymphocytes, etc, secrete it. It can be cleaved by proteases to form soluble homotrimers, or it can bind to multiple BAFF receptors to form polymers and thereby perform biological functions [24][25][26]. BAFF can promote the survival, maturation, proliferation and differentiation of B lymphocytes, induce the production of autoantibodies, facilitate the transformation of antibody categories, promote the secretion of inflammatory factors by T lymphocytes, and regulate the function of monocytes. Recent studies [27][28][29][30] have shown that BAFF is highly expressed in the serum of patients with idiopathic MN and is a key prognostic factor. The relevant analysis results indicate that the serum BAFF1 level of patients with positive anti-PLA2R antibodies is positively correlated with the anti-PLA2R antibody level. The serum BAFF level of MN patients was positively correlated with pathological stage (P<0.001). The results of the comparison of the serum BAFF levels among patients with different pathological stages were as follows: stage IV > stage III > stage II > stage I. Additionally, any pair's differences were statistically significant (P<0.05).

(1) Patients have primary MNs, which further activate the complement pathway in the body, aggravate damage to glomerular podocytes, lead to the secretion of different inflammatory mediators by lymphocytes, aggravate the body's condition, and increase serum BAFF levels.

(2) BAFF can participate in the proliferation of B lymphocytes and induce autoantibodies, further exacerbating the autoimmune inflammatory response.

CFB is a key factor in the complement replacement pathway, is mainly secreted and formed by liver cells and macrophages, and plays a role in the low-level complement replacement pathway. The complement C3 convertase cleaves complement C3 into C3a and C3b. Complement C3b can be converted into complement C3bBb under the action of CFB and complement factor H [31]. CFB can be used to assess immune function and the inflammatory state in various diseases [32]. Under the stimulation of various inflammatory mediators and factors, mononuclear macrophages and T lymphocytes can secrete MCP-1, which participates not only in the body's inflammatory response but also in processes such as cell proliferation, repair, and oxidative stress [33]. Previous studies [34][35][36] have shown that CFB and MCP-1 are associated with the prognosis of MN patients to some extent, but studies on the anti-PLA2R antibody and MN stage are rare. The levels of serum CFB and MCP-1 in patients gradually increased. Moreover, the correlation analysis indicates that serum CFB and MCP-1 levels in patients are positively correlated with anti-PLA2R antibody levels. Serum CFB and MCP-1 levels in MN patients were positively correlated with pathological stage, suggesting that these levels are closely associated with disease progression [37]. The bypass pathway can mediate and contribute to the development of MN, activate complement C3b generation in the early stage of the disease, and increase serum CFB levels. After complement C3b and CFB are converted to C3bBb, they can jointly activate the downstream complement pathway and promote immune complex formation, thereby damaging the podocyte basement membrane [38]. In addition, it releases large amounts of inflammatory factors, leading to a subsequent increase in serum MCP-1 levels. The more severe the podocyte and basement membrane damage in MN patients is, the weaker the glomerular filtration function becomes [39]. As the disease progresses, glomerular sclerosis, thickening of the renal vessel wall and renal interstitial fibrosis become more serious, further aggravating the patient's condition [40].

After 6 months of treatment in this study, the levels of serum anti-PLA2R antibodies, BAFF, CFB and MCP-1 in the nonremission group were greater than those in the remission group (P<0.05). Owing to the collinearity problem between the levels of serum BAFF, CFB and MCP-1 and anti-PLA2R antibodies, multivariate logistic regression analysis revealed that elevated levels of serum BAFF, CFB, and MCP-1 were risk factors for nonremission in MN patients after treatment (P<0.05), indicating that the higher the levels of serum BAFF, CFB, and MCP-1 in MN patients were, the more severe the condition was, and glomerular basement membrane hyperplasia was obvious. The filtration function shows progressive weakening. Some patients have no obvious relief or no relief at all after treatment, which affects their prognosis. The results of the ROC curve analysis revealed that the AUC of the combined prediction of serum BAFF, CFB and MCP-1 for the nonremission of MN patients after treatment was 0.948, which was significantly greater than that predicted by the anti-PLA2R antibody alone and the AUCs of BAFF, CFB and MCP-1 alone. These findings suggest that the combined detection of serum BAFF, CFB, and MCP-1 improves the prediction of nonremission in MN patients after treatment.

## Conclusion

Anti-PLA2R antibodies, MN stage, and treatment outcome are all correlated with serum levels of BAFF, CFB, and MCP-1 in MN patients. Serum BAFF, CFB, and MCP-1 have some value in predicting nonremission in MN patients after treatment, but their combined predictive value is greater. However, this study has several limitations. The levels of serum BAFF, CFB, and MCP-1 in MN patients were not dynamically monitored.

## Dodatak

### Acknowledgements

None.

### Funding

None.

### Conflict of interest statement

All the authors declare that they have no conflict of interest in this work.
